# Full-Genome Sequence of a Neuroinvasive West Nile Virus Lineage 2 Strain from a Fatal Horse Infection in South Africa

**DOI:** 10.1128/genomeA.00740-16

**Published:** 2016-07-28

**Authors:** Juliet L. D. Mentoor, Alison B. Lubisi, Truuska Gerdes, Stacey Human, June H. Williams, Marietjie Venter

**Affiliations:** a Zoonosis Research Unit, Department Medical Virology, University of Pretoria, Pretoria, South Africa; b Virology Molecular Epidemiology and Diagnostics Programme, Agricultural Research Council, Onderstepoort Veterinary Institute, Pretoria, South Africa; c Department of Paraclinical Sciences, Faculty of Veterinary Sciences, University of Pretoria, Pretoria, South Africa; d Centre for Global Disease Detection, South Africa, Division of Global Health Protection, Centers for Disease Control and Prevention, Pretoria, South Africa

## Abstract

We report here the complete genome sequence of a lineage 2 West Nile virus (WNV) strain that resulted in fatal neurological disease in a horse in South Africa. Several recent reports exist of neurological disease associated with lineage 2 WNV in humans and horses in South Africa and Europe; however, there are a lack of sequencing data from recent fatal cases in Southern Africa, where these strains likely originate. A better understanding of the genetic composition of highly neuroinvasive lineage 2 strains may facilitate the identification of putative genetic factors associated with increased virulence.

## GENOME ANNOUNCEMENT

West Nile virus (WNV) is a mosquito-transmitted flavivirus (family *Flaviviridae*) of global concern (1, 2). In humans, ~20% of infections result in WN fever associated with rash, arthralgia, and myalgia, while <1% may develop severe neurological disease and death (3). In horses, 20% of WNV cases exhibit clinical signs; however, ~90% of these cases may develop neurological disease, with a fatality rate approaching 30% (1). WNV lineage 2 (L2) viruses are endemic to South Africa and Madagascar (4, 5) and have emerged in Europe in the past 10 years, causing outbreaks in humans and horses (6, 7). WNV genome sequencing has provided insight into possible genetic factors that may differentiate between highly pathogenic and attenuated strains (8, 9). Previously, we described WNVL2 strains associated with severe neurological diseases in horses and humans in South Africa (4, 10–13). WNVL2 full-genome sequences from Africa are limited, with the only currently available sequence originating from a nonfatal encephalitis, a fatal hepatitis case, and febrile infections in humans from South Africa (8, 14).

As part of a study investigating WNV as the cause of severe neurological disease in horses in South Africa, an isolate (HS101/08) was obtained from a brain tissue sample from a horse that had died of severe neurological signs in 2008, including seizures and complete paralysis (4, 12). This study aimed to characterize this neuroinvasive strain by describing its complete genome sequence and comparing it to both highly and less neuroinvasive lineage 1 and 2 strains. HS101/08 was isolated on Vero cell monolayers, and supernatants were harvested after three passages. Viral particles were concentrated through 0.2-µm-pore microfilters (Sigma-Aldrich) and treated with DNase and RNase. Nucleic acid extraction was performed with the High Pure viral nucleic acid isolation kit (Roche Diagnostics, Mannheim, Germany) and the RNA minikit (Qiagen, Hilden, Germany). cDNA was synthesized with the Expand reverse transcriptase kit (Roche Diagnostics). Universal flavivirus and WNV-specific primers were used to amplify the complete genome (15). Cycle sequencing was performed with the ABI BigDye Terminator version 3.1 kit (Applied Biosystems, CA). Phylogenetic and nucleotide analyses were performed using BioEdit (version 7.0.5.2) and MEGA version 7 (16, 17).

The HS101/08 genome sequence was 11,042 bp in size and closely related to neuroinvasive strains SPU116/89 and SA93/01 (accession numbers EF429197 and EF429198) from South Africa and a subclade of neurovirulent human and horse strains from Italy and Greece (7, 8). HS101/08 shared 98% nucleotide identity with SPU116/89 and SA93/01. These two strains are highly neuroinvasive in mice (18, 19). An amino acid sequence comparison between HS101/08 and SA93/01 identified six uncharacterized substitution mutations predicted to have no effect on the structural or nonstructural proteins. We verified the presence of a 154N-S156 glycosylation site in the envelope protein sequences of South African L2 strains (including HS101/08), which were previously suggested to be associated with increased mouse neurovirulence (20). Similarities between the highly neurovirulent HS101/08 isolate and human isolates from cases of severe disease confirms that highly pathogenic L2 strains are still circulating in South Africa. WNV should be considered as a diagnosis in human and animal neurological cases.

### Nucleotide sequence accession number.

The accession number of the complete coding sequence of HS101/08 is JN393308.
